# Role of Endovascular Grafts in Combined Vascular and Skeletal Injuries of the Lower Extremity: a Preliminary Report

**DOI:** 10.5812/atr.10862

**Published:** 2013-06-01

**Authors:** Jon David Simmons, William Bryant Walker, Joseph William Gunter III, Naveed Ahmed

**Affiliations:** 1 Division of Trauma and Surgical Critical Care, University of South Alabama, Alabama, USA; 2 Section of Trauma and Surgical Critical Care, University of Mississippi Medical Center, Jackson, Mississippi, USA

**Keywords:** Endovascular Procedures, Stents, Bone Fractures, Wounds and Injuries

## Abstract

**Background:**

A gunshot wound to the lower extremity with combined skeletal and vascular injuries can be difficult to manage. In clinical practice, it is not always possible to assemble a multispecialty team to work seamlessly to attain this goal, and the end result may be unnecessary prolonged ischemic time. A covered endovascular stent (EVS) can be used initially to restore perfusion without need of a time-consuming temporary shunt in select cases.

**Objectives:**

The objective of this study is to compare novel methods of repairing the superficial femoral artery to the traditional three-step operative approach in patients that have a concomitant femur fracture.

**Patients and Methods:**

All patients with combined vascular and skeletal injuries to the lower extremity were reviewed retrospectively. Patients were divided into three groups: Group 1: EVS placed percutaneously. Group 2: EVS placed with a hybrid combination of open and endovascular technique. Group 3: Placement of temporary shunt followed by skeletal stabilization and definitive vascular repair.

**Results:**

There were 16 patients identified. Group 1 = three, Group 2 = six, Group 3 = seven. EVS can shorten time to revascularization and prevents a second episode of vascular interruption at the time of the final vascular repair. The structural stability of the EVS was strong enough to withstand the skeletal manipulation without deformation of the stent.

**Conclusions:**

EVS is comparable to open repair and has the strength to withstand orthopedic manipulation in the short term when used in combined vascular and skeletal injuries to the lower extremity. Ischemic time is reduced significantly if final revascularization is accomplished at the onset with an EVS and the process is more efficient if the trauma surgeon is able to repair the vascular injury. With increasing sophistication of endovascular devices, this offers an appealing approach to vascular injuries that will decrease ischemic and total operative times when compared to the more traditional three-stage repair.

## 1. Background

Gunshot wounds to the lower extremity with combined vascular and orthopedic injuries require a multi-disciplinary approach. This begins with a trauma surgeon providing the initial resuscitation and control of life threatening injuries. After control of significant bleeding, most trauma surgeons will place a temporary shunt to reconstitute distal perfusion during the skeletal stabilization of the leg. After the orthopedist provides skeletal stabilization, the trauma/vascular surgeon would then follow with definitive vascular repair (1). This three-step process requires tremendous coordination between specialists frequently resulting in a delay in definitive revascularization and prolonged operative times. We present an approach where the in-house trauma surgeon reconstitutes blood flow using a covered endovascular stent (EVS) as the initial and definitive vascular repair in selected patients. This eliminates the ischemic time during the third stage of the traditional repair and reduces total operative time in these complex trauma patients. In our limited experience, the placement of an EVS is faster and less technically demanding than a hand-sewn vascular graft (2). This is done by either a “Percutaneous” approach, where the arterial defect is bridged by a percutaneously placed EVS or a “Hybrid” approach that utilizes a limited open operation to facilitate the placement of a covered EVS across the injured artery. All EVS were intact after skeletal manipulation and left in place as permanent vascular conduits. Theoretically, the ischemia time can further be reduced if the vascular repair is done by the in-house trauma surgeon.

## 2. Objectives

In this study we attempt to compare this new approach (total percutaneous or hybrid) with the more “traditional” three-step approach. “Traditional” for our center, is vascular control, temporary shunt followed by final vascular repair after skeletal stabilization (1). We believe the percutaneous or hybrid operation will decrease total ischemic time by preventing the second episode of ischemia during the final vascular repair in the traditional approach.

## 3. Patients and Methods

We performed a retrospective chart review on all patients with an isolated gunshot wound to the leg with combined injuries to superficial femoral vessels and femur. This single center study was performed in a busy inner-city trauma center, located in Cleveland, Ohio, with a large volume of penetrating injuries from Jan 1, 2001 to Dec 31, 2005. The patients were stratified into three groups that are described below. Data abstracted included patient demographics and characteristics of the injuries and their management, which included total operative and ischemia times. The patients were placed in three groups for analysis.

**Figure 1.**
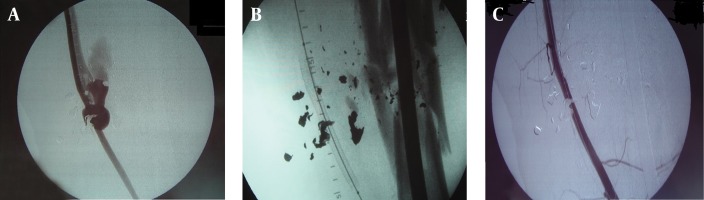
Preoperative On-table Angiogram A) Injured femoral artery with traumatic arterio-venous fistulae, B) Percutaneous endovascular stent placement, C) Angiogram after skeletal fixation. patent vessel with overlying shrapnel

**Figure 2.**
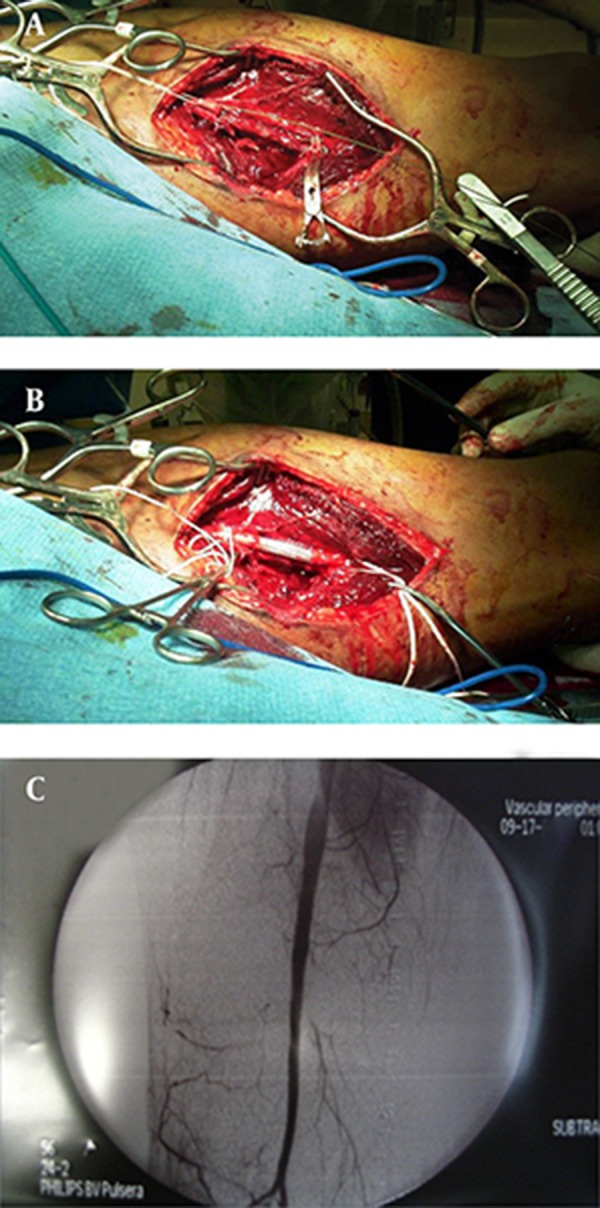
Approach Revealing a Missing Segment of Artery A, B) Hybrid Approach Revealing a Missing Segment of Artery, C) Completion Angiogram After Skeletal Fixation

Group 1: “Percutaneous operation” This group had a percutaneous placement of an EVS as the definitive vascular repair followed by skeletal stabilization. An arteriogram was performed in the operating room via a femoral artery exposure (Figure 1 A). A glide wire was then introduced from the ipsilateral femoral artery proximal control (hemostasis) was established by inflating a balloon occlusion catheter proximal to the injured superficial femoral artery (SFA). Attempt was then made to pass a glide wire across the injured segment. If successful and with favorable landing zones, a covered EVS (GOREVIABAHN^®^, Flagstaff, Arizona, USA) of appropriate size was percutaneously placed (Figure 1 B). An angiogram was done to confirm patency and repeated after skeletal fixation (Figure 1 C). Venous injuries could not be identified due to the nature of this procedure, unless there was a traumatic arterio-venous fistula (Figure 1 A). If an arterio-venous fistula was encountered the artery was repaired percutaneously with an EVS, the venous injuries were not repaired. Venogram was not done. Group 2: “Hybrid operation”: If the initial intra-operative angiogram from the femoral artery revealed a complete disruption due the loss of a segment of the SFA, a hybrid operation was performed. Proximal vascular control was achieved by inflating an endovascular balloon catheter over the glide wire in the proximal SFA, and both were left in place (similar to group 1). A limited incision was then utilized to access the missing segment of the artery. After exposing the injured segment of the SFA via a limited incision, a glide wire was introduced at the femoral artery and manually passed through the injury into the distal arterial segment (Figure 2 A). The EVS was then placed over the glide wire to bridge the missing arterial segment and deployed with a proximal and distal landing zone of approximately two centimeters (Figure 2 B). Again, a completion angiogram was done after skeletal stabilization (Figure 2 C). Venous injuries were repaired or ligated at the discretion of the surgeon. A glide wire has been threaded from the angiogram site in groin, exiting from the proximal arterial end of the missing segment. The glide wire was manually passed into the distal end of the missing arterial segment and the stent was placed from the groin incision. The stent can be clearly visualized bridging the missing segment of the artery.

**Figure 3.**
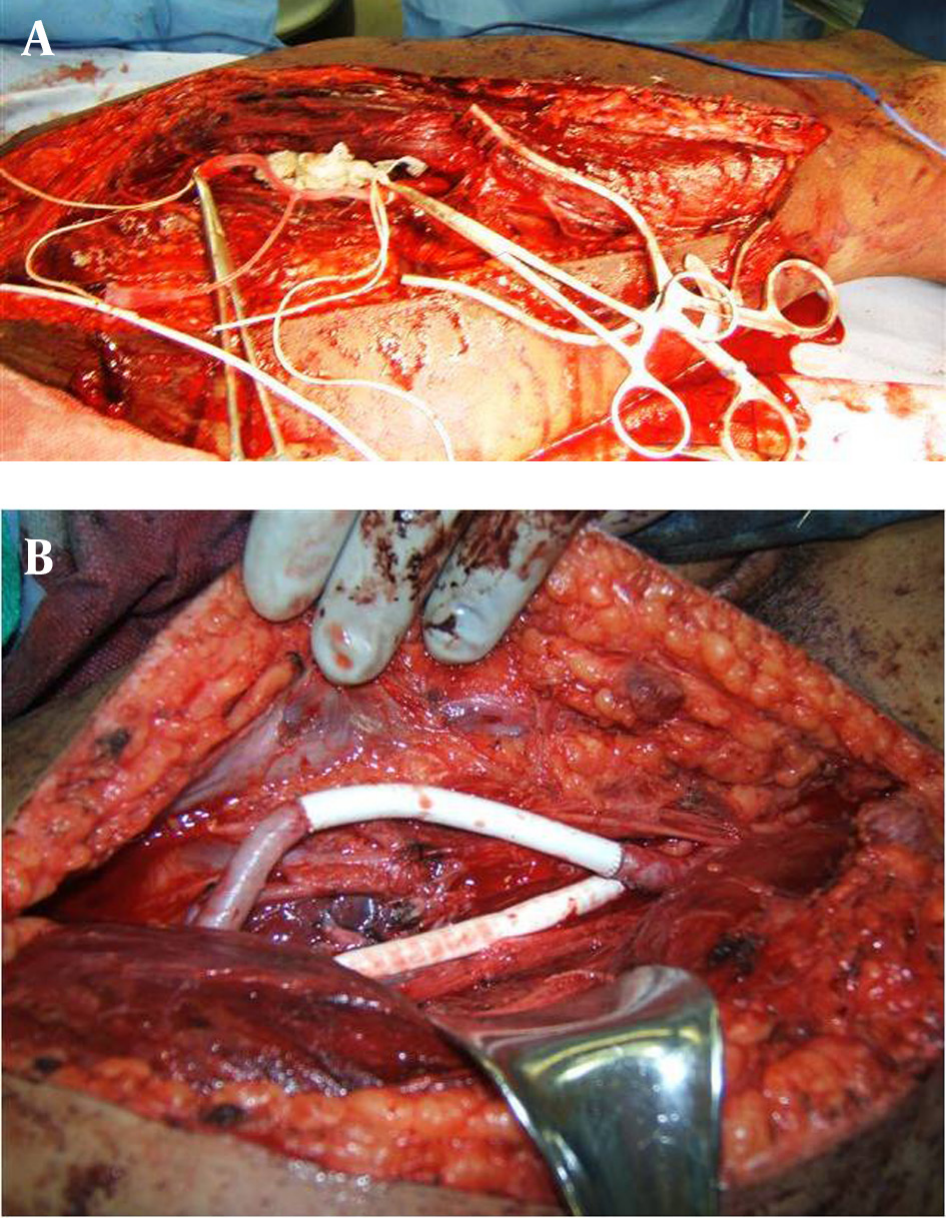
Treatment Approaches A) temporary shunt in the injured artery, B) final arterial and venous repairs after skeletal fixation

Group 3: “Traditional” three-step open operation: This involved a temporary vascular shunt, followed by skeletal fixation and final definitive vascular repair. Vascular control was achieved with an open operation, a temporary shunt was used to reconstitute blood flow, and definitive arterial repair was done with a polytetrafloroethylene (PTFE) graft after skeletal stabilization (Figures 3 A and Figure 3 B).

Venous injuries were repaired or ligated. Fasciotomy was performed if needed. All patients were discharged with 81 milligrams of Aspirin, and attempt was made to follow these patients at 3 weeks, 6 months and 1 year. All patients were placed on DVT prophylaxis in the immediate post operative period. Statistical analysis was performed using computer-assisted regression analysis and analyses of variance (ANOVA) were followed by unpaired t-tests for normally distributed data. For all others, we used non-parametric tests (Wilcox on Rank Sum) and nonparametric correlations (Spearman method). The statistical software, Sigma Stat for Windows (v.2, SPSS Science, Chicago, Ill) was the default program for all analyses. Statistical significance was defined at P < 0.05.

## 4. Results

A total of 16 patients were identified, all of which were male. Age and associated injuries with ischemia times are summarized in Tables 1 and Table 2 for all groups. Group 1 “Total Percutaneous”: Three patients had successful percutaneous placement of EVS. All were intact after orthopedic repair, and none of the patients required a fasciotomy. Total operative time was 29 ± 2.6 minutes. Time from arrival in emergency room to revascularization was 54.6 ± 6.0 minutes. Length of hospital stay was 8.6 ± 2.5 days. The only complication in this group was a pulmonary embolus following repair of traumatic arterial-venous fistula. Arterial EVS reestablished arterial flow to the injury. All repairs were patent at three month follow-up and there were no other short-term complications. All patients in this group were lost to further follow-up after 6 months. Group 2 “Hybrid”: Six patients had “Hybrid” placement of EVS. All stents were intact after orthopedic repair. Total operative time was 36.5 ± 7.2 minutes. Time to revascularization from arrival to emergency department was 73.6 ± 18.26 minutes; all had concomitant venous injuries that were ligated. A single patient required a fasciotomy in the post-operative period. Length of hospital stay was 15.1 ± 5.4 days. All stents were patent at three month follow-up, and one patient was followed for 2 years with no complications. Group 3 “Traditional three-step approach”: Seven patients had a traditional three-stage approach as previously described. All had concomitant arterial and venous injuries. All arterial and five venous injures were shunted temporarily and then repaired with a PTFE graft after skeletal fixation. Two venous injuries were ligated. Initial revascularization time was 68.8 ± 20.9 minutes. Arterial flow was further interrupted to remove the temporary shunt and to complete the definitive vascular repair, a “second hit” of 41.2 ± 22.1 minutes. Total ischemic time, the sum of the initial and second ischemic times, was 110.1 ± 41.4 minutes. Length of hospital stay was 15.1 ± 4.7 days (Table 2). All patients in this group received a fasciotomy. All patients had patent grafts at three months but then lost to follow-up. There were no wound or graft infections and no evidence of chronic venous insufficiency regardless of repair or ligation of the injured vein. Early complications (within fourteen days) included pulmonary emboli in 2 patients, both of which had a venous repair. There were no pulmonary emboli or venous insufficiency in patients that had a venous ligation. Statistical analysis of the data (Table 1 and Table 2) revealed that the three groups are similar in terms of demographics and initial ischemic time. However, the total ischemic time for group 3 was significantly longer when compared to groups 1 and 2 (P < 0.02). The difference in revascularization time between all the groups is also significant (P < 0.01). Length of hospital stay was significantly shorter for group 1 (P < 0.02), but there was no difference in length of stay between group 2 and 3. Using regression analysis, we found the only significant influence on length of stay is ischemic time. ANOVA revealed the total ischemia time was directly related to the technique used, group 1 was better than group 2 and group 2 was better than group 3. Nonparametric correlations (Spearman method) reinforced the significance of the correlations between ischemic time, revascularization time and hospital length of stay in all groups (P < 0.05).

**Table 1.** Characteristics of the Study Population

	Group 1 (n = 3), Mean ± SD	Group 2 (n = 6), Mean ± SD	Group 3 (n = 7), Mean ± SD
**Age, y**	25 ± 6.5	21 ± 4	27.1 ± 6.6
**Venous injuries**	1 ^a^	6	5
**Venous Ligation:Repair**		6:0	3:2
**Fasciotomy**	0	1	7

^a^Venous injuries could not be identified due to the nature of this procedure, one was found as patient had a traumatic arterio-venous fistula (Figure 1A), artery was fixed percutaneously, venous injury was not repaired and a venogram was not done.

**Table 2.** Time Associated with Each Method of Vascular Repair

	Group 1 (n = 3), Mean ± SD	Group 2 (n = 6), Mean ± SD	Group 3 (n = 7), Mean ± SD
**Ischemic time, min**	54.6 ± 6.0 ^a^	73.6 ± 18.26	78.8 ± 20.98
**Total ischemic time, min ** **^b^**	54.6 ± 6.0 ^a^	73.6 ± 18.26	110.0 ± 41.4^b^
**Operative time, min**	29 ± 2.6 ^a^	36.5 ± 7.2	39.7±9.2^c^62.0 ±19.8
**Length of stay, d**	8.6 ±2.5^a^^,^^c^	15.1±5.4	15±4.7

^a^P value < 0.05

^b^Total ischemic time in Group 3 has two components: Time from patient arrival to establishment of blood flow using a temporary shunt plus the “Second hit” time required to complete the final vascular repair. This excludes time limb was perfused while temporary shunt was in place.

^c^Operative time for Group 3 has two components (as above). These times represent arterial repair only. 1)Time from initial incision to blood flow (39.7 ± 9.2), 2)Time to complete the graft (62.0 ± 19.8)

## 5. Discussion

**Figure 4.**
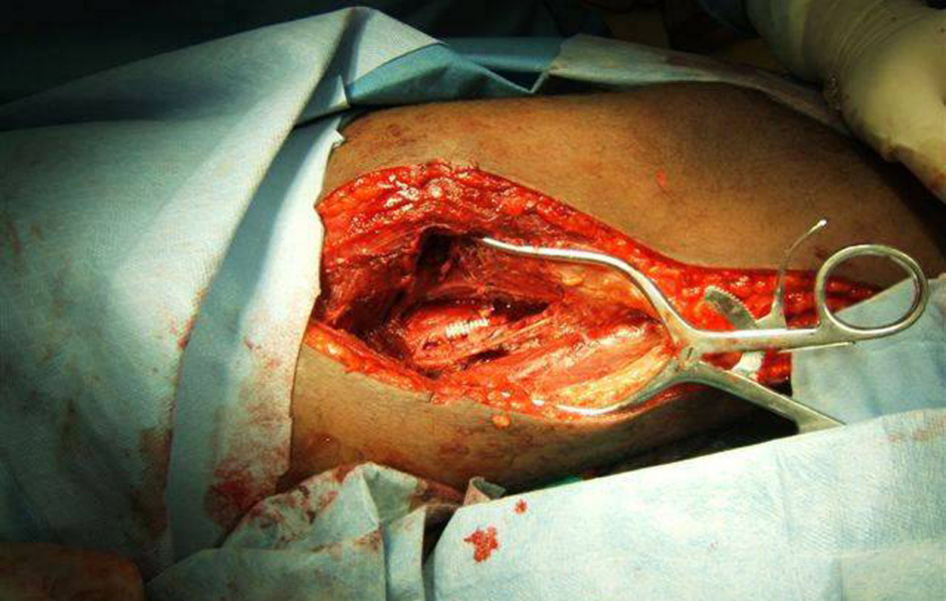
Endovascular Stent Placed With an Open Operation, With Limited Exploration. The stent can be clearly visualized bridging the missing segment of the artery.

Ischemia time is critical during the management of combined skeletal and vascular injuries in the lower extremity. The trauma surgeon must orchestrate the treatment with multiple surgeons that may be responding from home which increases the ischemic times. In this trauma center, trauma surgeons are in-house and primarily involved in all aspects of care, except skeletal fixation. The patient is immediately taken to the operating room where resuscitation is completed and the injury is localized with an on-table angiogram performed by the trauma surgeon. Traditionally, we have performed an open operation to establish blood flow with temporary vascular shunts in both artery and vein. The definitive vascular repair is performed by the trauma surgeon after skeletal stabilization using an appropriate sized PFTE vascular graft ( 2 - 4 ). Some centers have advocated performing the definitive vascular repair before skeletal fixation, thereby reducing the total ischemia time by avoiding the second episode of ischemia ( 5 ). However, this approach is not well-accepted for fear that tension on the suture line during skeletal manipulation may cause immediate disruption or development of a distal intimal flap. Our “percutaneous” and “hybrid” repair reduces ischemia time by performing the definitive vascular repair before skeletal fixation by utilizing EVS. In our opinion, EVS is more resilient than standard grafts ( 6 ). When the EVS was deployed percutaneously, ischemia time from emergency room to revascularization was significantly reduced. The overall length of procedure, need for a fasciotomy, and hospital length of stay were also significantly less when compared to our “traditional” approach. When the “Hybrid approach” was performed, ischemia time and length of operation was also significantly less when compared to the “traditional” three step approach. This hybrid approach does not require proximal and distal control of the artery. Therefore, with increasing experience we found this can be done with a much smaller incision (Figure 4). This is a novel way to bridge a missing segment of artery efficiently with minimal dissection (Figure 2 B). The only post operative complication seen were pulmonary emboli (one in Group 1 and two in Group 3). The only recognized venous injury in group one, an arteriovenous fistula, developed a post-operative pulmonary embolism. Two of the three venous injuries that were repaired in group 3 also developed pulmonary emboli. Interestingly, none of the ligated veins developed this complication. There are several limitations to our study. This study possesses the same limitations of any retrospective analysis. It represents only one institution’s experience and the infrequent occurrence of these injuries prevents adequate sample size for adequate subgroup analysis. The study population and the retrospective nature of this study limit the ability of long-term follow-up. Although this is a retrospective review of our experience, with negligible long-term follow-up, it does provide an alternate method for treatment of this complicated injury. With increasing sophistication in endovascular technology, this will provide trauma surgeons with an easier and faster method to repair these injuries. We have shown that the trauma surgeon can place EVS with safety and efficiency in the emergency setting with excellent short-term results. The long-term affects of EVS in patients with these injuries is not known but even if the stent becomes occluded at a later date, the patient will be more tolerant of distal ischemia due to a better physiologic state in a non-trauma situation. We do caution the trauma surgeon to receive proper training with endovascular techniques before attempting EVS in the trauma patient. EVS is comparable to open repair and has the strength to withstand orthopedic manipulation when used in combined vascular and skeletal injuries to the lower extremity. Ischemic time is reduced significantly when final revascularization is accomplished at the onset and the process is more efficient if the trauma surgeon is able to repair the vascular injury. With increasing sophistication of endovascular devices, this offers an alternate approach to vascular injuries that will decrease ischemic and total operative times when compared to the more traditional three-stage repair.
